# Preclinical Evaluation of Caprylic Acid-Fractionated IgG Antivenom for the Treatment of Taipan (*Oxyuranus scutellatus*) Envenoming in Papua New Guinea

**DOI:** 10.1371/journal.pntd.0001144

**Published:** 2011-05-17

**Authors:** Mariángela Vargas, Alvaro Segura, María Herrera, Mauren Villalta, Ricardo Estrada, Maykel Cerdas, Owen Paiva, Teatulohi Matainaho, Simon D. Jensen, Kenneth D. Winkel, Guillermo León, José María Gutiérrez, David J. Williams

**Affiliations:** 1 Instituto Clodomiro Picado, Facultad de Microbiología, Universidad de Costa Rica, San José, Costa Rica; 2 School of Medicine and Health Sciences, University of Papua New Guinea, Port Moresby, Papua New Guinea; 3 Australian Venom Research Unit, University of Melbourne, Parkville, Australia; 4 Nossal Institute for Global Health, University of Melbourne, Parkville, Australia; Liverpool School of Tropical Medicine, United Kingdom

## Abstract

**Background:**

Snake bite is a common medical emergency in Papua New Guinea (PNG). The taipan, *Oxyuranus scutellatus*, inflicts a large number of bites that, in the absence of antivenom therapy, result in high mortality. Parenteral administration of antivenoms manufactured in Australia is the current treatment of choice for these envenomings. However, the price of these products is high and has increased over the last 25 years; consequently the country can no longer afford all the antivenom it needs. This situation prompted an international collaborative project aimed at generating a new, low-cost antivenom against *O. scutellatus* for PNG.

**Methodology/Principal Findings:**

A new monospecific equine whole IgG antivenom, obtained by caprylic acid fractionation of plasma, was prepared by immunising horses with the venom of *O. scutellatus* from PNG. This antivenom was compared with the currently used F(ab')_2_ monospecific taipan antivenom manufactured by CSL Limited, Australia. The comparison included physicochemical properties and the preclinical assessment of the neutralisation of lethal neurotoxicity and the myotoxic, coagulant and phospholipase A_2_ activities of the venom of *O. scutellatus* from PNG. The F(ab')_2_ antivenom had a higher protein concentration than whole IgG antivenom. Both antivenoms effectively neutralised, and had similar potency, against the lethal neurotoxic effect (both by intraperitoneal and intravenous routes of injection), myotoxicity, and phospholipase A_2_ activity of *O. scutellatus* venom. However, the whole IgG antivenom showed a higher potency than the F(ab')_2_ antivenom in the neutralisation of the coagulant activity of *O. scutellatus* venom from PNG.

**Conclusions/Significance:**

The new whole IgG taipan antivenom described in this study compares favourably with the currently used F(ab')_2_ antivenom, both in terms of physicochemical characteristics and neutralising potency. Therefore, it should be considered as a promising low-cost candidate for the treatment of envenomings by *O. scutellatus* in PNG, and is ready to be tested in clinical trials.

**Author Summary**

Snake bite envenoming represents an important public health hazard in Papua New Guinea (PNG). In the southern lowlands of the country the majority of envenomings are inflicted by the taipan, *Oxyuranus scutellatus*. The only currently effective treatment for these envenomings is the administration of antivenoms manufactured in Australia. However, the price of these products in PNG is very high and has steadily increased over the last 25 years, leading to chronic antivenom shortages in this country. As a response to this situation, an international partnership between PNG, Australia and Costa Rica was initiated, with the aim of generating a new, low-cost antivenom for the treatment of PNG taipan envenoming. Horses were immunised with the venom of *O. scutellatus* from PNG and whole IgG was purified from the plasma of these animals by caprylic acid precipitation of non-immunoglobulin proteins. The new antivenom, manufactured by Instituto Clodomiro Picado (Costa Rica), was compared with the currently available F(ab')_2_ antivenom manufactured by CSL Limited (Australia). Both were effective in the neutralisation of the most relevant toxic effects induced by this venom, although the whole IgG antivenom showed a higher efficacy than the F(ab')_2_ antivenom in the neutralisation of the coagulant activity.

## Introduction

Envenoming by snake bite is a common medical emergency in Papua New Guinea (PNG) [1]–[3]. Despite incomplete epidemiological data, studies in PNG show that the incidence of snake bite ranges from under five cases per 100,000 people per year in the mountains of Goilala and Hiri (Central Province) and in Madang, to 526–561 cases per 100,000 people per year in the coastal Kairuku lowlands [1], [2], [4]. A mortality rate of 7.9 deaths per 100,000 people per year in Central Province was reported for the period 1987–1992 [2]. At Port Moresby General Hospital (PMGH) only envenomed snakebite patients are admitted, and most of these are sent to the Intensive Care Unit (ICU). A study of snakebite admissions to the PMGH ICU between 1992 and 2001 revealed case fatality rates of 8.2% for adults and 14.6% for children [5]. More recently, case fatality rates of 14.5% for adults and 25.9% for children have been reported from the ICU of the same hospital [3].

**Figure 1:**
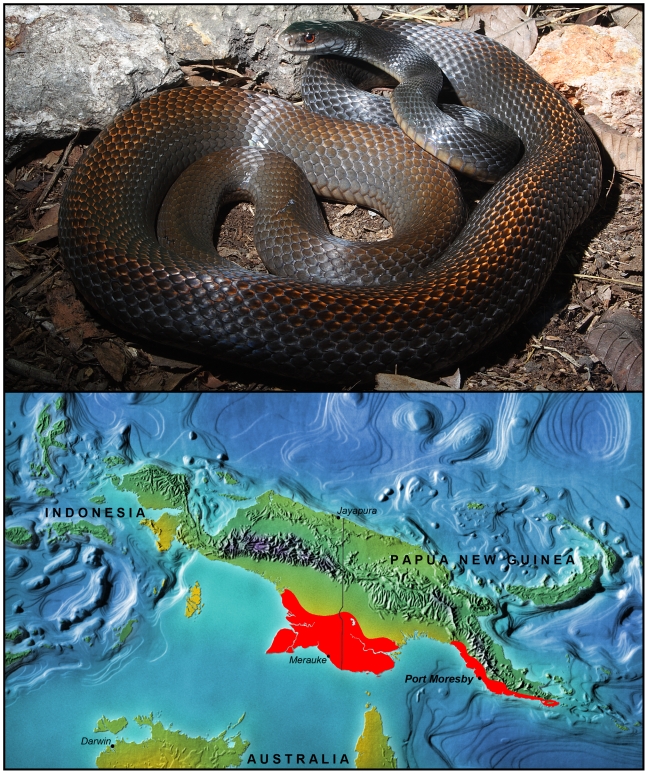
*Oxyuranus scutellatus* from Papua New Guinea. Adult specimen from Padi Padi, Milne Bay Province, Papua New Guinea, and distribution map showing the range of this species in PNG and Indonesia's Papua Province (Photo and artwork: DJ Williams).

Throughout PNG three species of elapid snakes are responsible for nearly all systemic envenomings: *Acanthophis laevis* (smooth-scaled death adder), *Micropechis ikaheka* (New Guinea small-eyed snake), and *Oxyuranus scutellatus* (Papuan taipan). A very small number of envenomings are caused by other *Acanthophis* species, *Pseudechis papuanus* (Papuan blacksnake) and *Pseudonaja textilis* (New Guinea brownsnake) [3]. For many years the Papuan taipan has been regarded as a separate subspecies (*Oxyuranus s. canni*) to Australian populations (*Oxyuranus s. scutellatus*). However, recent taxonomic and biogeographical studies have shown that, despite some perceived morphological differences, molecular genetic analysis reveals no significant differentiation between the two populations [6], [7]. On this basis, *O. scutellatus* is now considered a single species with both Australian and New Guinean populations. In southern PNG and neighbouring southern Papua, up to 95% of life-threatening snake bites are caused by *O. scutellatus* (Fig 1). The effects of taipan bite include mild local effects and severe systemic manifestations characterised by coagulopathy with spontaneous systemic haemorrhage, myotoxicity, irreversible flaccid paralysis, acute kidney injury and cardiac disturbances [2], [3], [8]–[10]. The neurotoxic manifestations of taipan bite are dominated by the effects of extremely potent, destructive, presynaptic phospholipase A_2_ toxins, resulting in physical damage to nerve terminals [11], [12]. Only the early (within 4–6 hours) administration of suitable antivenom can prevent or reduce this presynaptic damage; consequently, when treatment is delayed, severe paralysis occurs, requiring endotracheal intubation and mechanical ventilation until neuromuscular synapses have regenerated [2], [13].

Intravenous administration of either taipan monospecific antivenom or polyvalent antivenom prepared in Australia by CSL Limited (CSL) against the venom of Australian *O. scutellatus*, has been the only effective treatment for envenomings by *O. scutellatus* in PNG [2], [3], [14]. *In vitro* preincubation studies, using chick biventer cervicis preparations, have shown that this antivenom inhibits the neurotoxic effects of *O. scutellatus* venom sourced in Indonesian Papua [15], and clinical observations in PNG have shown its effectiveness in halting spontaneous systemic bleeding and restoring blood coagulability [2], [14]. Administration of antivenom within four hours of envenoming significantly reduces the incidence of respiratory paralysis [2]. Therefore, a critical issue concerning the management of *O. scutellatus* envenoming in PNG is the need for rapid access to antivenom, which in turn demands its widespread distribution to hospitals and other health centres. One critical factor limiting the availability of CSL antivenom in PNG is its high price, which has increased more than 800% over the last two decades [5], [16], greatly reducing the capacity of the health system to purchase adequate volumes to meet all of the country's needs, leading to chronic shortages. As a consequence, a thriving black market in stolen antivenoms has developed, with private sellers charging as much as US$3,200.00/vial [17].

An alternative method for manufacturing antivenoms is based on the purification of horse IgG antibodies by fractionation of hyperimmune plasma with caprylic acid [18]–[21]. This simple and inexpensive procedure yields a highly purified whole IgG preparation [19]. Polyspecific antivenoms prepared in this way, at relatively low cost, have been tested in clinical trials in Colombia [22]–[24] and Nigeria [25], and displayed excellent efficacy and safety. We describe a collaborative effort between teams in Costa Rica, Australia and PNG in the preparation and preclinical evaluation of a monospecific whole IgG antivenom against the venom of *O. scutellatus* from PNG, and have compared it with the Australian-made CSL taipan antivenom currently in use.

## Methods

### Venom

A pool of two grams of venom was obtained from twelve healthy, adult specimens of *O. scutellatus* collected in PNG's Milne Bay Province and Central Province. These snakes were maintained in a purpose-built serpentarium at the University of PNG, and venom was collected at 21 day intervals. Venom was obtained using Parafilm-covered 50 mL Eppendorf tubes. Samples contaminated by blood were discarded, and all samples were handled using plastic pipettes and tubes. Venom was snap-frozen to −80°C, before being freeze-dried and stored away from light at −20°C. In some experiments, the venom of Australian *O. scutellatus* obtained from Venom Supplies Pty Limited (Tanunda, South Australia) was used for comparative purposes. In all experiments, lyophilized venom was dissolved in 0.14 M NaCl, 0.04 M phosphate, pH 7.2 (PBS) immediately before use.

### Antivenoms

Two antivenoms were used in this study:

- (a). Monospecific taipan antivenom manufactured by CSL Limited (CSL), Melbourne, Victoria, Australia (batch B0548-06301; expiry date March 2012).
- (b). Monospecific taipan antivenom manufactured by Instituto Clodomiro Picado (ICP) (batch 4511209 ICP; expiry date November 2012).

### Antivenom Production

CSL monospecific taipan antivenom is prepared from the plasma of horses immunised with the venom of *O. scutellatus* from Australia. It is manufactured using a protocol based on pepsin digestion and ammonium sulphate fractionation of plasma, and therefore consists of F(ab')_2_ fragments [26]. ICP monospecific taipan antivenom is raised in horses immunised with the venom of *O. scutellatus* from PNG. Immunisation was performed in a group of three horses which had not previously been used for antibody production, by using Freund's complete and incomplete adjuvants during the first two injections, respectively, followed by subsequent inoculations of venom dissolved in PBS. All injections were performed by the subcutaneous route in a single anatomical site. When a satisfactory neutralising titre was reached, the animals were bled from the jugular vein, with the blood being collected in 6 L plastic bags containing sodium citrate as anticoagulant. After sedimentation of blood cells, the plasma was separated and the immunoglobulins purified by caprylic acid precipitation (5% final concentration of caprylic acid and one hr stirring) [19]. After filtration in 8 µm pore filter paper, the filtrate was diafiltered and then formulated to contain 7.5 g/L NaCl, 1.6 g/L phenol, pH 7.2 (Table 1). The antivenom solution was sterile filtered using 0.22 µm pore membranes and glass vials were filled with 40 mL of antivenom. The resultant antivenom met all the requirements of the quality control protocol at Instituto Clodomiro Picado.

**Table 1:** Physicochemical characteristics of antivenoms.

*Characteristic*	*ICP IgG antivenom*	*CSL F(ab')_2_ antivenom*
Protein (g/L)	45.9±0.9	144.6±0.4
Phenol (g/L)	1.6±0.04	2.12±0.03
pH	7.2	6.4
Caprylic acid (mg/L)	31±1	−
Monomer content (%)^a^	93±1.0	93±0.4
Turbidity (NTU)^b^	25	23

a Monomer content is expressed as the percentage of antivenom protein present as either IgG or F(ab')_2_ monomers, as analyzed by gel filtration (see Materials and Methods).

b Turbidity is expressed as Nephelometric Turbidity Units (see details in Materials and Methods).

### Physicochemical analysis of antivenoms

Total protein concentration was determined by the Biuret test [27], and electrophoretic analysis was performed by SDS-PAGE, under non-reducing conditions, using an acrylamide concentration of 7.5% [28]. Gels were stained with Coomassie Brilliant Blue R-250. Phenol concentration was determined according to a modification of the method of Lacoste et al. [29]. Caprylic acid concentration was quantified by HPLC according to Herrera et al. [30]. The content of antibody monomers was assessed by FPLC gel filtration in a Superdex 200 10/300 GL column using 0.15 M NaCl, 20 mM Tris, pH 7.5, as eluent. Turbidity was determined using a turbidimeter (La Motte, model 2020, Chestertown, MD), and expressed as nephelometric turbidity units (NTU).

### Toxic and enzymatic activities of venoms and neutralisation by antivenoms

#### Ethics statement

The experimental protocols involving the use of animals in this study were approved by the Institutional Committee for the Care and Use of Laboratory Animals (CICUA) of the University of Costa Rica, and adhere to the International Guiding Principles for Biomedical Research Involving Animals of the Council of International Organizations of Medical Sciences (CIOMS).

#### Lethal activity

Groups of 5 CD-1 mice of both sexes were injected via either the intraperitoneal (i.p.; 16–18 g mice) or the intravenous (i.v.; 18–20 g mice) routes with various amounts of PNG *O. scutellatus* venom, dissolved in PBS. The volume of injection was 0.5 mL when using the i.p. route and 0.2 mL when using the i.v. route. Deaths occurring during 48 hr were recorded and the Median Lethal Dose (LD_50_) was estimated by Spearman-Karber [31].

#### Myotoxic activity

Groups of 4 CD-1 mice (18–20 g) of both sexes were injected intramuscularly (i.m.), in the right gastrocnemius, with various amounts of venom dissolved in 50 µL PBS. Control mice received 50 µL PBS under otherwise identical conditions. After three hr, mice were bled from the tail and the blood was collected into heparinised capillary tubes. After centrifugation, plasma was collected and the creatine kinase (CK) activity of plasma was quantified by using a commercial kit (CK-Nac, Biokon Diagnostik, Germany). The Minimum Myotoxic Dose (MMD) corresponds to the dose of venom that induced an increment in plasma CK activity corresponding to four times the CK activity of mice injected with PBS [32].

#### Coagulant activity

The method described by Theakston and Reid [33] and Gené et al. [34] was followed. Various amounts of venom, dissolved in 100 µL of 0.15 M NaCl, were added to aliquots of 200 µL of human citrated plasma previously incubated at 37°C for 5 min. Experiments were run in quadruplicate. Clotting times were recorded, and the Minimum Coagulant Concentration (MCC) was determined. The MCC corresponds to the concentration of venom that induced plasma clotting in 60 sec. Experiments were performed in both citrated plasma and in citrated plasma to which CaCl_2_ was added (15 µL of 0.2 M CaCl_2_ to 200 µL plasma) immediately before addition of the venom, as previously described [35], [36].

#### Phospholipase A_2_ (PLA_2_) activity

PLA_2_ activity was determined titrimetrically, using egg yolk phospholipids as substrates, as previously described [37]. Activity was expressed as µEq fatty acid released per mg protein per min.

#### Neutralisation of venom activities by antivenoms

A fixed venom dose (“challenge dose”) was pre-incubated with various dilutions of antivenom prior to testing in the corresponding experimental systems, as previously described [32], [38]. The following challenge doses of venom were used: Lethality (four venom LD_50_s); coagulant effect (two venom MCCs); myotoxicity (one venom MMD since higher doses were lethal); PLA_2_ activity (1.5 µg venom). For each effect, the challenge dose of venom was incubated with various dilutions of antivenom, in order to achieve several ratios of mg venom/mL antivenom. Controls included venom incubated with PBS, or 0.15 M NaCl in the case of coagulant effect, instead of antivenom. Incubations were performed for 30 min at 37°C and then aliquots of the mixtures, containing the challenge dose of venom, were tested in the relevant assay systems described above. In the case of coagulant effect, mixtures of venom and antivenom used in the coagulation assay were incubated for no more than 3 min at room temperature (22–25°C) before testing, because incubation of venom for 30 min resulted in a partial loss of coagulant activity (see below). The neutralising ability of antivenoms for lethal and PLA_2_ activities were expressed as Median Effective Dose (ED_50_), corresponding to the ratio mg venom/mL antivenom in which the activity of the venom was reduced by 50% [38]. In the case of the coagulant effect, neutralisation was expressed as Effective Dose (ED), corresponding to the ratio mg venom/mL antivenom in which the clotting time is prolonged three times as compared with the clotting time of plasma incubated with venom alone [34]. On the other hand, the estimation of the neutralizing potency against myotoxicity could not follow the method previously described [32], since the challenge dose used (1 µg) induced a relatively small increment in plasma CK, and higher doses were lethal. Thus, the increment in plasma CK activity in control mice injected with 1 µg of venom alone was only 4 times the value of CK in mice injected with PBS. In these conditions, the value of ED was defined as the ratio mg venom/mL antivenom in which the plasma CK activity was not significantly different from the plasma CK activity of mice injected with PBS alone, i.e. where myotoxicity was completely abrogated.

### Variation of venom activities upon incubation

Pre-incubation of PNG *O. scutellatus* venom at 37°C for 30 min resulted in a significant loss of coagulant activity. Thus, it was considered that degradation of the procoagulant toxins may be occurring. On this basis, all of the venom activities assessed in this study (LD_50_, MCC, MMD and PLA_2_) were determined for venom solutions (a) immediately after dissolution in PBS and (b) after 30 min of incubation at 37°C. Lethality (LD_50_) by both i.p. and i.v. routes, myotoxicity (MMD) and PLA_2_ activity were not affected by incubation at 37°C for 30 min. In contrast, the coagulant activity of *O. scutellatus* venom was reduced upon incubation at 37°C for 30 min. We therefore altered the experimental design of the ED-MCC assay by limiting incubation of venom and antivenom to not more than 3 minutes, in order to avoid the loss of activity associated with incubation.

### Statistical analysis

All descriptive statistic calculations and the Mann Whitney U test used to determine the significance of the differences between the median values of two non-parametric experimental groups in the neutralisation tests were performed using the InStat statistics program.

## Results

### Characteristics of antivenoms

**Figure 2:**
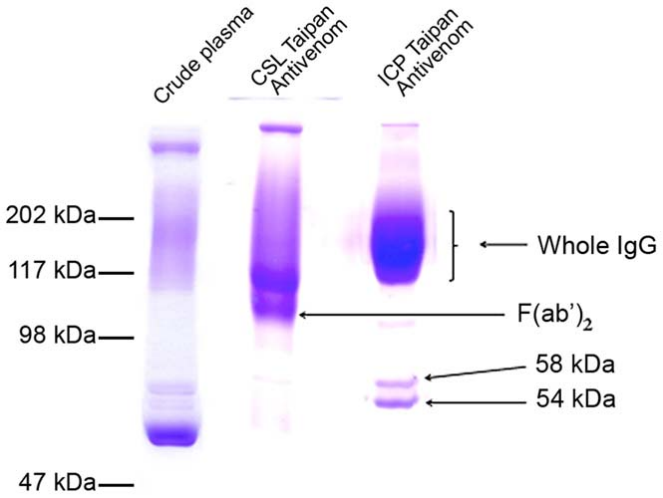
Electrophoretic analysis of antivenoms. Non-reduced samples were loaded in a 7.5% polyacrylamide gel in the presence of SDS. After separation, proteins were stained with Coomassie Brilliant Blue R-250. Migration of molecular mass markers is depicted to the left.

**Figure 3:**
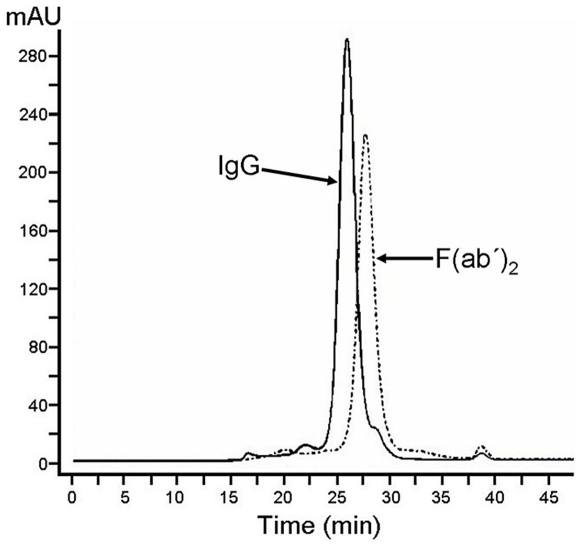
Analysis of antivenoms by gel filtration. Aliquots of the antivenoms were separated by gel filtration in Superdex 200 10/300 GL column and elution was carried out with 150 mM NaCl, 20 mM Tris-HCl, pH 7.5 buffer. Both antivenoms showed a major peak, corresponding to either F(ab')_2_ or IgG monomers, which comprise >90% of the total protein.

The physicochemical characteristics of the two antivenoms are summarised in Table 1. The CSL F(ab')_2_ antivenom had a 3.15 fold higher total protein concentration than ICP whole IgG antivenom. When examined using SDS-PAGE run under non-reducing conditions, the CSL antivenom, manufactured by pepsin digestion and ammonium sulphate fractionation, presented predominant bands in a molecular mass range corresponding to F(ab')_2_ fragments (Fig 2). In contrast, the whole IgG antivenom fractionated with caprylic acid showed one predominant band of a molecular mass corresponding to IgG monomers. Both antivenoms presented additional minor bands of high molecular mass (in the case of F(ab')_2_ antivenom) and of 54 and 58 kDa (in the case of IgG antivenom) (Fig 2). Both antivenoms were composed by >90% IgG or F(ab')_2_ monomers, as judged by gel filtration analysis (Table 1; Fig 3), thus showing a very low protein aggregate content, and had very low turbidity, as assessed both visually and through quantification by nephelometry (Table 1).

### Toxic and enzymatic activities of venom and neutralising profile of antivenoms

#### Toxic and enzymatic activities of *O. scutellatus* venom

The venom of *O. scutellatus* is highly toxic for CD-1 mice, with LD_50_ values of 0.04±0.01 µg/mouse (2.3±0.6 µg/kg) and 0.08±0.01 µg/mouse (4.2±0.5 µg/kg) for the i.p. and the i.v. routes, respectively. Lethality was associated with neurotoxicity, as mice showed evidence of limb and respiratory paralysis. Venom also caused myotoxicity in mice, with a MMD of 1 µg per mouse, corresponding to the dose that increased 4 times the plasma CK activity as compared with mice injected with PBS, which had a CK activity of 187±34 U/L. Mice receiving doses higher than 1 µg died before 3 hr. *O. scutellatus* venom induced coagulation of human plasma, with higher activity in conditions in which CaCl_2_ was added to citrated plasma before the addition of the venom, as previously reported [35], [36]. Without addition of calcium, the MCC was 0.76±0.20 µg/mL, whereas when CaCl_2_ was added to plasma immediately before the venom, the MCC was 0.33±0.13 µg/mL (p<0.05). The PLA_2_ activity of this venom corresponds to 297±7 µEq fatty acid released per mg protein per min (Table 2).

**Table 2:** Toxic activities of *O. scutellatus* venom and neutralisation by antivenoms.

*Effect*	*Activity*	*ICP IgG antivenom (Batch 4511209 ICP)*		*CSL F(ab')_2_ antivenom (Batch B0548*-*06301)*	
		mg Venom/mL Antivenom^d^	Neutralising Units^e^	mg Venom/mL Antivenom^d^	Neutralising Units^e^
Lethality (i.p.) (LD_50_)^a^	0.04±0.01 µg	4.50 (3.18–6.41)	18,000	5.65 (3.89–8.77)	18,645
Lethality (i.v.) (LD_50_)^a^	0.08±0.01 µg	4.35 (3.05–5.29)	17,400	5.81 (4.08–7.04)	19,173
Coagulant (MCC)^b^ without calcium	0.76±0.20 µg/mL	2.43±0.29*		0.84±0.04*	
Coagulant (MCC) with calcium	0.33±0.13 µg/mL	2.37±0.08*		0.45±0.17*	
Myotoxic (MMD)^c^	1 µg	4.0		4.0	
PLA_2_ ( µEq/mg/min)	297±7	1.47±0.29		1.10±0.38	

a Lethal activity was determined for the i.p. and i.v. routes in CD-1 mice. Median Lethal Dose (LD_50_) is expressed as µg venom/mouse (mean ± S.D)

b Coagulant activity, expressed as the Minimum Coagulant Concentration (MCC), either in citrated plasma or in citrated plasma to which CaCl_2_ was added immediately before the test. Results are the mean ± S.D.

c Myotoxic activity, expressed as the Minimum Myotoxic Dose (MMD).

d Neutralisation is expressed as either ED_50_ (lethality and PLA_2_) or ED (coagulant and myotoxic effects) (see Materials and Methods for details). Results are presented as mg venom neutralised per mL antivenom. For lethality, the 95% confidence limits are included in parenthesis. For the other effects, results are presented as mean ± S.D. (n = 4–6). Challenge doses of venom correspond to 4 LD_50_s (lethality), 2 MCCs (coagulant), 1 MMD (myotoxicity), and 1.5 µg venom (PLA_2_ activity).

e CSL taipan antivenom is labelled as containing at least 12,000 Neutralising Units of antivenom, where 1 Unit = 0.01 mg venom neutralised (i.e.: 12,000 units neutralises 120 mg venom). Actual neutralising unit values for each antivenom are given here for comparison and are calculated from fill volumes of each product.

*: p<0.05 when the two antivenoms are compared.

#### Neutralisation by antivenoms

The two antivenoms were effective at neutralising the four activities tested. No significant differences were observed in the ED_50_ of either product against the lethal and PLA_2_ activities of PNG *O. scutellatus* venom as well as in the ED against myotoxic effect (Table 2). In contrast with lethal, PLA_2_ and myotoxic activities, there was a significant difference in the value of ED against *in vitro* coagulant activity of the venom of *O. scutellatus* from PNG, with the ICP antivenom having 5.2 times and 2.9 times the potency of the CSL antivenom, in experiments performed with calcium and without calcium, respectively (Table 2). In order to ascertain whether this difference is due to antigenic variations between the procoagulants of the venoms from the Australian and Papuan populations of *O. scutellatus*, the neutralisation of coagulant activity of the venom of Australian *O. scutellatus,* which is used in the immunizing mixture of the CSL antivenom, was investigated. The MCC of this venom was 1.3±0.6 µg/mL (in experiments where calcium was added to plasma) and 4.1±0.6 µg/mL (without added calcium) (p<0.05). Both antivenoms effectively neutralized this activity, with ICP antivenom showing a higher potency. The EDs in conditions where calcium was added were 10.0±2.5 mg venom neutralized per mL (ICP antivenom) and 4.1±1.4 mg venom/mL antivenom (CSL antivenom) (p<0.05). On the other hand, when calcium was not added to plasma, the values of EDs were 4.9±0.4 mg venom/mL antivenom (ICP antivenom) and 1.5±0.2 mg venom/mL antivenom (CSL antivenom) (p<0.05). When comparing the neutralizing ability of the antivenoms against all effects studied, since the CSL antivenom contains 3.15 times the protein of the ICP antivenom, a lesser amount of antivenom protein is required to achieve neutralisation of the various effects in the case of ICP antivenom.

## Discussion

In the present work, a new, whole IgG monospecific antivenom, obtained by caprylic acid precipitation, was prepared against the venom of Papua New Guinean *O. scutellatus*, the most medically important venomous snake in the southern halves of both PNG and Indonesian Papua. It was shown that, in standard WHO-endorsed preclinical neutralisation assays, against venom of *O. scutellatus* from PNG, this new antivenom compares very favourably with the F(ab')_2_ taipan antivenom currently in use in Australia and PNG.

The venoms of *Oxyuranus* spp. are among the most toxic ever reported [39], and our LD50 data on mice confirm the high toxicity of *O. scutellatus* from PNG. This high toxicity is due predominantly to the presence of a number of neurotoxins, in particular the presynaptic PLA_2_ trimer, taipoxin (“cannitoxin”) which destroys nerve terminals and also binds to skeletal muscle, leading to myolysis [11], [40]–[42]. A number of monomeric neurotoxic and myotoxic PLA_2_, a 52 kDa multimeric voltage-dependent calcium channel blocker, taicatoxin, and a 6.7 kDa post-synaptic α-neurotoxin of 6.7 kDa, α-oxytoxin 1, have also been characterized [43]–[47]. The dominance of destructive presynaptic neurotoxicity in the clinical syndrome of *O. scutellatus* envenoming has important implications for the treatment of taipan bites, since anticholinesterases do not improve neurotransmission, and more importantly antivenom cannot reverse established neurotoxic manifestations secondary to physical damage to nerve terminals [2], [48]. Both antivenoms tested in this work are effective in the neutralisation of the lethal effect of Papuan *O. scutellatus* venom after pre-incubation, thus evidencing their capacity to neutralize the neurotoxins present in this venom.

Disruption of the integrity of skeletal muscle fibre plasma membranes, with rapid impairment of the ability of this membrane to regulate its permeability to ions and macromolecules, is induced by the myotoxic PLA_2_s, taipoxin and OS2, from the venom of *O. scutellatus* [42], [49]–[50]. Both of the tested antivenoms effectively neutralised myotoxicity due to *O. scutellatus* venom. Since the neurotoxic and myotoxic actions of taipoxin, and similar presynaptically-acting neurotoxins, depend on PLA_2_ enzymatic activity of these toxins [51], [52], the effectiveness of the two antivenoms to neutralize PLA_2_ activity of *O. scutellatus* venom is compatible with the neutralisation of these toxic activities.

Coagulopathy occurs in a majority of patients envenomed by *O. scutellatus* in PNG [8]. This is due to the procoagulant effect of serine proteinases that are potent prothrombin activators [53]–[55]. In agreement with previous studies [35]–[36], Papuan *O. scutellatus* venom showed higher *in vitro* coagulant activity on plasma in conditions where CaCl_2_ was added to plasma immediately before venom. Regardless of whether the experiments were performed with or without the addition of calcium, ICP antivenom showed a higher potency for the neutralisation of coagulant effect. Interestingly, ICP antivenom was also more effective in the neutralisation of coagulant activity induced by venom from Australian *O. scutellatus.* These observations suggest that the differences shown by these antivenoms regarding neutralisation of coagulant effect are not likely to be due to antigenic variations in the procoagulant enzymes of these two populations of taipan, but instead to a higher antibody titre against these enzymes of both venoms in ICP antivenom. The basis of this finding remains to be elucidated, but may have to do with differences in the immunization schemes employed in the production of these antivenoms. It is recommended that the analysis of the neutralisation of coagulant activity by Australian venoms by antivenoms should be performed in conditions where calcium is added to citrated plasma before the addition of venom, for reasons previously described [35]–[36]. Clinical studies carried out in PNG demonstrated that CSL taipan antivenom was effective at restoring blood coagulability within 6–12 hr in 93% of patients treated [8]. A future clinical trial will determine whether this difference in the neutralisation of *in vitro* coagulant activity between these antivenoms will translate into differences in their *in vivo* clinical efficacy at restoring blood coagulability in envenomed patients.

An unexpected observation during this study was the partial loss of *in vitro* coagulant activity of the venom upon incubation at 37°C for 30 min. Whether this observation is due to proteolytic degradation or alteration in the quaternary structure of the procoagulant present in this venom, or to a physiologically sub-optimal environment in the *in vitro* coagulant activity assay remains unknown. Notably, serine proteinase inhibitors had to be used in the isolation of the prothrombin activator [54] and an apparent *in vivo* inactivation of the coagulant activity of *O. scutellatus* venom was described in a monkey model of envenoming [56]. Consequently, we modified the coagulant activity neutralisation protocol in order to avoid the incubation of venom at 37°C for 30 min. Although this modification departs from the conventional way to assess neutralization by antivenoms, i.e. incubation of venom-antivenom mixtures for 30 min, a shorter incubation time, such as the one adapted in this study, also allows a proper testing of neutralization of this activity, since the binding of antibodies to antigens is a very rapid phenomenon. Moreover, both antivenoms were able to neutralize coagulant activity in these circumstances.

There were two important considerations in embarking upon a project that is designed to develop an alternative to a currently available product of established efficacy. These were:

- (a). Antivenom price.

From 1987 to 2007 the cost of CSL polyvalent and taipan antivenoms to the Papua Guinea Department of Health increased drastically, leading to a 40% decline in product availability [57], [58]. As a consequence, these antivenoms have become increasingly unaffordable to a health system already under enormous stress, leading to chronic antivenom shortages of antivenom and negative patient outcomes [16]. The high prices and relative scarcity have led to a flourishing black market, where stolen antivenoms are resold by private pharmacies and unlicensed wholesalers [17]. We have focused on the need to produce an effective antivenom with a fill volume (40–50 mL) sufficient to neutralize the average “milked” venom yield (120 mg) of healthy, adult *O. scutellatus*, at the lowest sustainable price, as a means of restoring access to affordable antivenom supplies.

- (b). Local capacity-building.

PNG currently lacks the capacity to produce its own antivenoms or vaccines. Our successful collaborative development of a potent experimental Papuan taipan antivenom demonstrates the relevance of international partnership for approaching public health issues. This project has allowed the development of PNG capacity for venomous snake husbandry, and production of venoms for immunization and quality control. Further efforts will be aimed at strengthening other local capacities in PNG which, in the long term, may lead to the sustainable manufacture of antivenoms in this country.

In conclusion, a new low-cost whole IgG antivenom, obtained by caprylic acid fractionation of horse plasma, was prepared against the venom of *O. scutellatus* from PNG. The antivenom has a satisfactory preclinical profile in the neutralisation of lethal, PLA_2_, myotoxic and coagulant effects of *O. scutellatus* venom, comparable to that of the F(ab')_2_ antivenom currently in use in PNG. These two antivenoms will be compared further in a randomised, non-inferiority, controlled trial in PNG in order to determine the clinical efficacy and safety profiles of both products.
